# Breast Lesions Diagnosed as Ductal Carcinoma *In Situ* by Ultrasound-Guided Core Needle Biopsy: Risk Predictors for Concomitant Invasive Carcinoma and Axillary Lymph Node Metastasis

**DOI:** 10.3389/fonc.2021.717198

**Published:** 2021-09-10

**Authors:** Yanbiao Liu, Xu Wang, Ang Zheng, Xinmiao Yu, Zining Jin, Feng Jin

**Affiliations:** Department of Breast Surgery, The 1st Affiliated Hospital, China Medical University, Shenyang, China

**Keywords:** core needle biopsy, underestimation, risk predictor, ductal carcinoma *in situ*, platelet

## Abstract

**Background:**

The major concern over preoperatively diagnosed ductal carcinoma *in situ* (DCIS) of breast *via* ultrasound-guided core needle biopsy (US-CNB) is the risk of missing concomitant invasive carcinoma. It is crucial to identify risk predictors for such a phenomenon and evaluate its impact on axillary conditions to help surgeons determine which patients should receive appropriate axillary lymph node management.

**Methods:**

Medical records of 260 patients preoperatively diagnosed with DCIS *via* 14-gauge CNB were retrospectively analyzed. All of them underwent subsequent surgery at our institution and were successively divided into invasive and non-invasive groups, and metastatic and non-metastatic groups according to pathology of resected specimens and metastasis of axillary lymph nodes (ALNs). Predictive value of preoperative physical examinations, imaging findings, histopathological findings, and hematological indexes for pathological underestimation and metastasis of ALN was assessed by logistic regression analysis.

**Results:**

The concomitant invasive carcinoma was overlooked in 75 out of 260 patients (29.3%). Multivariate analysis revealed that presence of microinvasion, presence of abnormal lymph node on ultrasound, and absent linear or segmental distributed calcification on mammography were independent risk predictors for invasive carcinoma. Fourteen patients had lymph node metastasis, and five of them were in the non-invasive group. The presence of abnormal lymph node on ultrasound and increased ratio of platelet distribution width to platelet crit (PDW/PCT) (>52.85) were identified as independent risk predictors for ALN metastasis.

**Conclusion:**

For patients diagnosed with DCIS preoperatively, appropriate ALN management is necessary if they have risk predictors for concomitant invasive carcinoma and ALN metastasis.

## Introduction

Ductal carcinoma *in situ* (DCIS) refers to proliferating neoplastic epithelial cells that are confined to the ductal system of breast (1). It is acknowledged as a precursor lesion to most, if not all, invasive breast cancers (2). Currently, DCIS accounts for approximately 25% of all breast cancer cases diagnosed each year, owing to the remarkable advances in breast imaging tools over the past few decades (3). Clinically, ultrasound-guided core needle biopsy (US-CNB) has become a routine method to diagnose DCIS with advantages of a higher level of accuracy, less trauma, and lower expense as compared with open biopsy (4). However, despite its advantages, patients preoperatively diagnosed with DCIS *via* US-CNB may be confirmed with invasive carcinoma postoperatively due to sampling error, which is a congenital deficiency of this technology (5).

The uncertainty of this phenomenon, so-called pathological underestimation, leads to a dilemma in surgical decision-making (6). For patients with concomitant invasive carcinoma, appropriate axillary lymph node (ALN) management allows for an accurate assessment of the axillary condition and provide guidance for subsequent regional and systemic treatments (7). In contrast, for those with pure DCIS, such an operation seems to be redundant, as the indolent nature and enclosed microenvironment of DCIS cells dictate their inability to migrate to local lymph nodes, let alone distant organs (8).

In clinical settings, patients are routinely recommended to receive ALN management at the same time as breast surgery. This crude surgical decision, although avoiding the possibility of a secondary operation that could cause more damage to patients, indisputably results in a waste of medical resources and economic cost. Therefore, it is crucial to clarify risk predictors for pathological underestimation to formulate a more accurate surgical plan preoperatively (9).

Some features of tumor have been shown to correlate with pathological underestimation, such as presence of comedo-necrosis, palpability of lesions, lesion size on mammography, and suspected microinvasion (5), in addition to a hematological index platelet–lymphocyte ratio (PLR), which has also been identified as a risk predictor for pathological underestimation (10). However, none of these predictors were universally acknowledged due to the differences in sample population and technical conditions among medical centers in various studies.

In the present study, we retrospectively analyzed medical records of 260 patients preoperatively diagnosed with DCIS *via* US-CNB; compared differences in clinicopathological features between invasive and non-invasive, and metastatic and non-metastatic groups; and explored underlying relationships among them, with an attempt to investigate risk predictors for concomitant invasive carcinoma and ALN metastasis.

## Methods

### Patients

From June 2012 to December 2020, 530 patients were diagnosed with DCIS *via* US-CNB at the First Affiliated Hospital of China Medical University. After careful screening, data for patients who were reported to have ipsilateral or contralateral invasive breast carcinoma or did not undergo subsequent surgery at our institution were excluded. It was worth noting that two patients were diagnosed with concurrent DCIS in breast and ALN metastasis *via* US-CNB preoperatively, which we believed was due to the presence of undetected invasive carcinoma in the lesion. They both chose to receive neoadjuvant chemotherapy first and, thus, were not included in this study. Two hundred sixty patients in total were finally enrolled. All patients had complete resection of lesions and were successively divided into invasive and non-invasive groups, and metastatic and non-metastatic groups according to pathology of resected specimens and metastasis of ALNs for further analysis.

### Risk Predictors

Based on a review of existing studies (11–13), we analyzed predictive value of preoperative physical examinations, imaging findings, histopathological findings, and hematological indexes for pathological underestimation and metastasis of regional lymph nodes using logistic regression analysis. The following factors were evaluated: age; menstruation status; family history of cancer; patient-reported symptoms including palpability, nipple discharge, and pain; number of punctual strips; presence and grading of mass on ultrasound; presence of abnormal lymph node on ultrasound; presence of linear or segmental distributed calcification on mammography; maximal size of lesions; grading of DCIS; presence of microinvasion; DCIS subtype papillary; immunohistochemical indexes including estrogen receptor (ER), progesterone receptor (PR), HER2, and Ki-67; and hematological indexes including platelet distribution width (PDW), PDW to platelet crit ratio (PDW/PCT), neutrophil–lymphocyte ratio (NLR), PLR, and systemic immune infiltration index (SII). Ultrasound and mammograms were performed by appropriate professional teams, and all reports were reviewed and signed by qualified specialists. Grading of lesions was on the basis of Breast Imaging Reporting and Data System (BI-RADS). The maximal size of lesions was measured on ultrasound because of its high sensitivity and accuracy to masses. In multifocal lesions, the sum of maximum diameters of the whole pathological area was calculated.

### Biopsy Method

All enrolled patients were with masses identified on ultrasound and recommended for core needle biopsy.

Two seasoned radiologists carried out all biopsies with a 14-gauge semi-automated core needle under the guidance of ultrasound. A minimum of four punctual strips were taken if the lesion was of sufficient size. The entire procedure was supervised by an attending radiologist to confirm accuracy.

### Pathological Diagnosis

All biopsy samples were routinely fixed in 4% neutral buffered formalin and embedded in paraffin before being cut into 4-μm-thick sections and histopathologically analyzed with hematoxylin and eosin staining. Further immunohistochemical studies were performed in cases where definitive diagnosis of stroma invasion was difficult. DCIS was classified as low, intermediate, or high grade on the basis of nuclear grading, taking into account necrosis, caryokinesis, and histomorphology changes (14). Papillary subtype referred to the finger-like protrusion under light microscopy. Based on the criteria of the American Joint Committee on Cancer (AJCC), microinvasion was defined as migration of a portion of tumor cells across basement membrane into periductal stroma, yet the maximum size of invasive foci is equal to or less than 1 mm (15). Receptor positive for ER and PR was defined as more than 1% of tumor cell nuclei that tested positive (16). Expression of HER2 and Ki-67 was also graded. Expression of HER2 was classified as 0, 1+, 2+, and 3+ levels according to percentage of cells with positively stained cell membrane to all cells (17, 18). As for expression of Ki-67, 30% was regarded as the threshold to distinguish between high and low levels (19, 20).

### Blood Sample Collection

Peripheral blood samples were obtained within 1 week prior to biopsy. We focused on PDW, PCT, PDW/PCT, PLR, NLR, and SII. PLR was calculated as platelet/lymphocyte. NLR was calculated as neutrophil/lymphocyte. And SII was calculated as neutrophil × platelet/lymphocyte.

### Statistical Analysis

Pearson’s chi-square test and continuous correction chi-square test were used for statistical differences among categorical variables. Student’s t-test was used to compare the difference between means of two groups of continuous variables, conforming to a normal distribution, with the results presented in the form of mean ± standard deviation (SD). The Mann–Whitney U test was used to test for hierarchical variables and continuous variables that did not conform to a normal distribution. Logistic regression models were used to determine independent predictors. Variables with *p* < 0.1 in univariate analysis were included in subsequent multivariate analysis. *p* < 0.05 was considered statistically significant. All statistical analyses were carried out using SPSS version 26.0.

## Results

### Underestimation of Invasiveness

Seventy-five out of 260 patients were diagnosed with invasive carcinoma postoperatively. The pathological underestimation rate of US-CNB was 29.3% in this study.

### Clinicopathological Characteristics

Clinicopathological characteristics were compared between patients in both groups with results shown in **Table 1**. The difference in mean age between the two groups was not statically significant (53.21 ± 10.78 *vs*. 52.84 ± 11.37, *p* = 0.807). A higher percentage of patients in the invasive group had left-sided carcinoma as compared with those in the non-invasive group (62.67% *vs*. 50.00%, *p* = 0.064). No statistically significant differences were noticed in terms of tumor size (3.9 *vs*. 3.5 cm, *p* = 0.123), palpability (*p* = 0.210), and pain (*p* = 0.257). Twelve patients (6.49%) in the non-invasive group complained of nipple discharge, while only one patient (1.33%) in the invasive group suffered from the same symptom. To our surprise, no statistically significant differences were found in number of puncture strips between the two groups (*p* = 0.993). It was worth mentioning that we noted a higher and statistically significant proportion of patients with microinvasion in the invasive group than in the non-invasive group (22.67% *vs*. 5.41%, *p* < 0.001). No statistically significant differences in other pathological parameters between the two groups were noticed. When it came to surgery, more patients in the non-invasive group underwent breast-conserving surgery (BCS) compared with those in the invasive group (9.73% *vs*. 8.00%), but the difference showed no statistical significance (*p* = 0.684). Metastasis of ALN was reported in 14 out of 257 patients who received ALN management, of whom nine patients belong to the invasive group and five patients belong to the non-invasive group, with statistically significant difference (*p* = 0.006).

**Table 1:** Characteristics between invasive and non-invasive groups.

Characteristics	Invasive group (n = 75)	Non-invasive group (n = 185)	*p*-Value
Age (n = 260)	53.21 ± 10.78	52.84 ± 11.37	0.807
Anatomic neoplasm subdivisions (n = 259)			
Left	47 (18.1%)	92 (35.5%)	
Right	28 (10.8%)	92 (35.5%)	0.064
Menstruation (n = 260)			
Menopause	44 (16.9%)	82 (31.5%)	
Pre-menopause	31 (11.9%)	103 (39.6%)	0.036
Maximal size (n = 255)	3.9 (2.8, 5.8)	3.5 (2.5, 5)	0.123
Palpability (n = 260)			
Palpable	66 (25.4%)	151 (58.1%)	
Impalpable	9 (3.5%)	34 (13.1%)	0.210
Pain (n = 260)			
Painful	29 (11.2%)	58 (22.3%)	
Painless	46 (17.7%)	127 (48.8%)	0.257
Discharge of papilla (n = 260)			
Present	1 (0.4%)	12 (4.6%)	
Absent	74 (28.5%)	173 (66.5%)	0.158
Number of puncture strips (n = 259)	4 (3, 6)	4 (3, 5)	0.993
Status of microinvasion (n = 260)			
Present	17 (6.5%)	10 (3.8%)	
Absent	58 (22.3%)	175 (67.3%)	<0.001
Grade of DCIS (n = 182)			
Low	5 (2.7%)	29 (15.9%)	
Intermediate	11 (6.0%)	20 (11.0%)	
High	36 (19.9%)	81 (44.5%)	0.122
DCIS subtype papillary (n = 260)			
Present	5 (1.9%)	5 (1.9%)	
Absent	70 (26.9%)	180 (69.2%)	0.250
ER status (n = 253)			
Positive	43 (17.0%)	113 (44.7%)	
Negative	27 (10.7%)	70 (27.7%)	0.963
PR status (n = 253)			
Positive	47 (18.6%)	116 (45.8%)	
Negative	23 (9.1%)	67 (26.5%)	0.577
HER2 status (n = 251)			
0	6 (2.4%)	9 (3.6%)	
1+	14 (5.6%)	31 (12.4%)	
2+	20 (8.0%)	55 (21.9%)	
3+	29 (11.6%)	87 (34.7%)	0.621
Ki-67 (n = 254)			
Low (30%)	62 (24.4%)	163 (64.2%)	
High (>30%)	8 (3.1%)	21 (8.3%)	0.997
Surgery of breast (n = 259)			
Mastectomy	68 (26.3%)	167 (64.5%)	
BCS	6 (2.3%)	18 (10.8%)	0.684
Metastasis of LN (n = 257)			
Present	9 (3.5%)	5 (1.9%)	
Absent	64 (24.9%)	179 (69.6%)	0.006

p value < 0.05 indicates statistical significance.

DCIS, ductal carcinoma in situ; ER, estrogen receptor; PR, progesterone receptor; BCS, breast-conserving surgery; LN, lymph node.

### Risk Predictors for Invasiveness

Results of logistic regression analysis are listed in **Table 2**. In univariate analysis, menstruation status (*p* = 0.037), linear or segmental distributed calcification on mammography (*p* < 0.001), and microinvasion (*p* < 0.001) were demonstrated as independent risk predictors for invasiveness. Abnormal lymph node on ultrasound was marginally significant (*p* = 0.071). However, no hematological indexes were demonstrated as independent risk predictors. Further multivariate analysis showed that abnormal lymph node on ultrasound (odds ratio, 2.832; confidence interval, 1.407–1.700; *p* = 0.004), linear or segmental distributed calcification on mammography (odds ratio, 0.05; confidence interval, 0.017–0.146; *p* < 0.001), and microinvasion (odds ratio, 23.5; confidence interval, 5.997–52.097; *p* < 0.001) were all independent risk predictors for invasive carcinoma.

**Table 2:** Risk predictors for invasiveness.

Factors	Univariate analysis		Multivariate analysis	
	OR (95% CI)	*p*-Value	OR (95% CI)	*p*-Value
Age	1.003 (0.979, 1.027)	0.706		
Menstruation (menopause *vs*. pre-menopause)	1.783 (1.035, 1.070)	0.037	1.751 (0.870, 3.524)	0.117
Family history of cancer (with *vs*. without)	1.213 (0.860, 1.710)	0.432		
Number of puncture strips	1.009 (0.894, 1.138)	0.890		
Palpability (palpable *vs*. impalpable)	1.651 (0.750, 1.637)	0.213		
Nipple discharge (present *vs*. absent)	0.185 (0.025, 1.526)	0.119		
Pain (painful *vs*. painless)	1.380 (0.789, 1.414)	0.258		
BI-RADS of mass on US (Grade 5 *vs*. Grade 4c *vs*. Grade 4b *vs*. Grade 4a)	1.225 (0/843, 1.779)	0.287		
Abnormal lymph node on us (present *vs*. absent)	2.195 (0.935, 5.151)	0.071	5.758 (1.708, 19.406)	0.005
Linear/segmental calcification (present *vs*. absent)	0.13 (0.063, 0.266)	<0.001	0.043 (0.014, 0.132)	<0.001
Pathological grade (high *vs*. intermediate *vs*. low)	1.603 (1.059, 1.424)	0.133		
Maximal size of lesion	1.065 (0.959, 1.184)	0.24		
Microinvasion (present *vs*. absent)	5.129 (2.224, 11.830)	<0.001	32.580 (7.916, 134.087)	<0.001
DCIS subtype papilloma (present *vs*. absent)	2.571 (0.722, 9.156)	0.145		
ER (positive *vs*. negative)	0.987 (0.560, 1.738)	0.963		
PR (positive *vs*. negative)	1.180 (0.659, 2.113)	0.577		
HER2 (3+ vs. 2+ vs. 1+ vs. 0)	0.828 (0.617, 1.110)	0.207		
Ki-67 (>30% *vs*. ≤30%)	1.002 (0.422, 2.379)	0.997		
PDW	0.971 (0.827, 1.140)	0.723		
PDW/PCT	1.001 (0.984, 1.018)	0.923		
SII (>600 *vs*. ≤600)	1.250 (0.620, 2.519)	0.533		
PLR (>160 *vs*. ≤60)	1.337 (0.704, 2.538)	0.374		
NLR (>150 *vs*. ≤150)	1.024 (0.546, 1.919)	0.941		

DCIS, ductal carcinoma in situ; ER, estrogen receptor; PR, progesterone receptor; PDW, platelet distribution width; PCT, platelet crit; SII, systemic immune infiltration index; PLR, platelet–lymphocyte ratio; NLR, neutrophil–lymphocyte ratio.

### Correlation of Preoperative Factors With Axillary Lymph Node Metastasis

**Figure 1:**
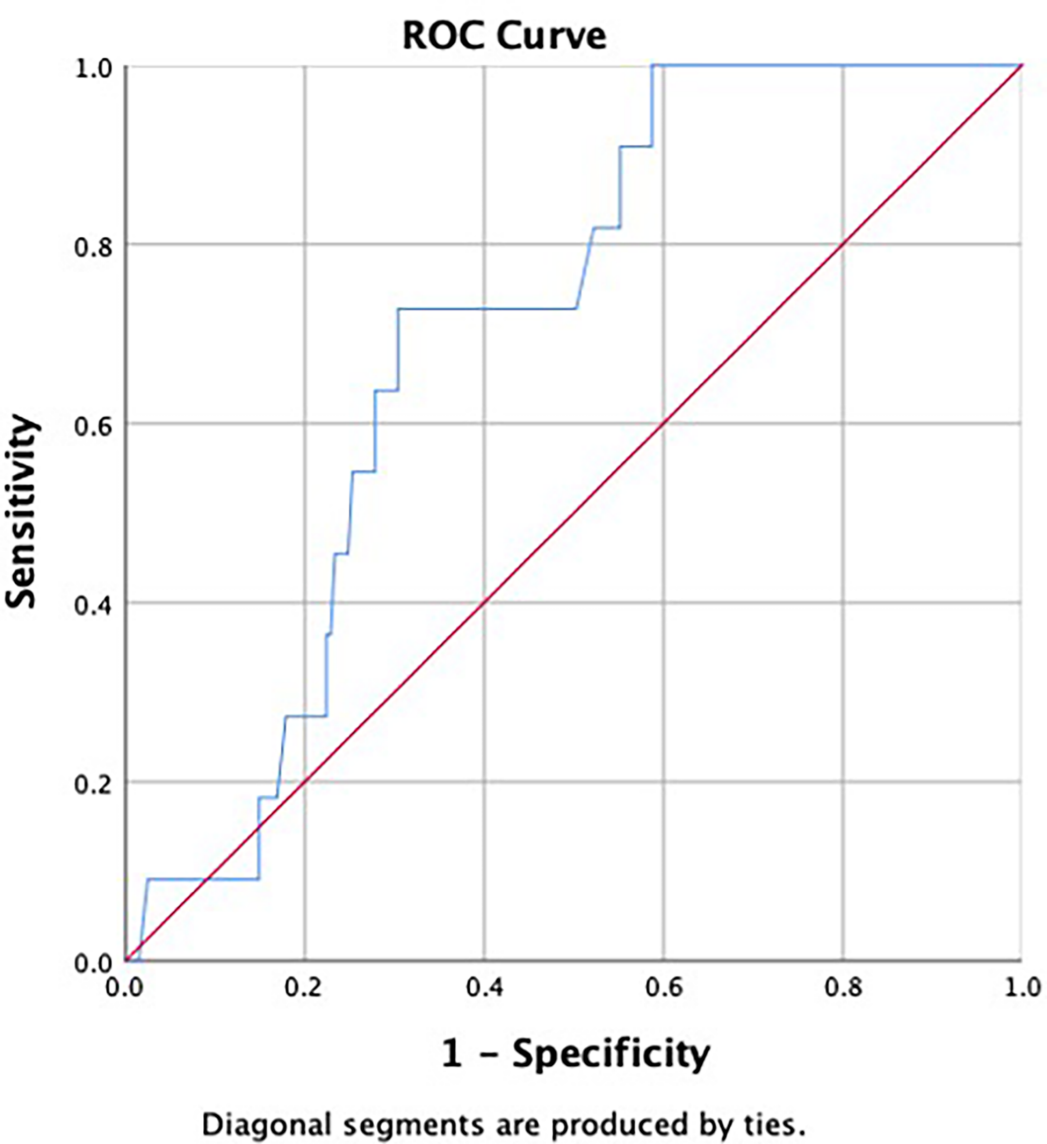
Receiver operating characteristic (ROC) curve was used to assess discrimination power of ratio of platelet distribution width to platelet crit (PDW/PCT). The area under the curve (AUC) was 0.701.

None of the patients were palpated with enlarged ALN on physical examination. To analyze the correlation of preoperative imaging results, histopathological findings, and hematological indexes with ALN metastasis, we first investigated their differences between patients in the metastatic and non-metastatic groups. The results are shown in **Table 3**. Differences with statistical significance were noticed in terms of PDW/PCT (*p* = 0.025) and abnormal lymph node on ultrasound (*p* < 0.001). Then, receiver operating characteristic (ROC) analysis was performed to determine the cutoff value and assess discriminative power of PDW/PCT. The cutoff value was 52.85 (corresponding to the maximal Youden index), and the area under the curve (AUC) was 0.701 (**Figure 1**). After univariate and multivariate regression analyses, high expression of PDW/PCT (>52.85) (odds ratio, 5.354; confidence interval, 1.246–23.001; *p* = 0.024) and abnormal lymph node on ultrasound (odds ratio, 6.894; confidence interval, 1.310–31.264; *p* = 0.023) were clarified as independent risk predictors for ALN metastasis. The results are shown in **Table 4**.

**Table 3:** Characteristics between metastatic and non-metastatic groups.

Characteristics	Metastatic group(n=14)	Non-metastatic group(n=243)	*P* value
Status of microinvasion (n=257)			
Present	2 (0.8%)	25 (9.7%)	
Absent	12 (4.7%)	218 (84.8%)	0.979
Grade of DCIS (n=257)			
Low	1 (0.4%)	33 (12.8%)	
Intermediate	2 (0.8%)	29 (11.3%)	
High	4 (1.6%)	111 (43.2%)	0.741
ER status (n=251)			
Positive	4 (1.6%)	150 (59.8%)	
Negative	8 (3.2%)	89 (35.5%)	0.082
PR status (n=251)			
Positive	5 (2.0%)	156 (62.2%)	
Negative	7 (2.8%)	83 (33.1%)	0.175
Her2 status (n=249)			
0	0 (0%)	15 (6.0%)	
1+	4 (1.6%)	41 (16.5%)	
2+	3 (1.2%)	71 (28.5%)	
3+	5 (2.0%)	110 (44.2%)	0.399
Ki-67 (n=251)			
Low ()	2 (0.8%)	27 (10.8%)	
High (>30%)	10 (4.0%)	212 (84.5%)	0.916
Abnormal lymph node on US (n=251)			
Present	6 (2.4%)	17 (6.8%)	
Absent	7 (2.8%)	221 (88.0%)	<0.001
Linear/segmental calcification (n=225)			
Present	3 (1.3%)	104 (46.2%)	
Absent	9 (4.0%)	109 (48.4%)	0.108
Maximal size of lesion (n=257)	4.65 (3.45,6.13)	3.60 (2.50,5.01)	0.077
PDW (n=257)	12.70 (11.80,14.20)	12.20 (11.10,13.30)	0.288
PDW/PCT (n=257)	57.62 (44.33,64.00)	44.67 (38.00,58.06)	0.025
SII (n=257)	362.78 (349.85,536.23)	412.47 (305.59,539.50)	0.792
PLR (n=257)	119.40 (95.15,159.76)	128.68 (101.28,165.11)	0.619
NLR (n=257)	2.19 (1.40,2.64)	1.68 (1.42,2.16)	0.285

DCIS, ductal carcinoma in situ; ER, estrogen receptor; PR, progesterone receptor; PDW, platelet distribution width; PCT, platelet crit; SII, systemic immune infiltration index; PLR, platelet–lymphocyte ratio; NLR, neutrophil–lymphocyte ratio.

**Table 4:** Risk predictors for axillary lymph node metastasis.

Factors	Univariate analysis		Multivariate analysis	
	OR (95% CI)	*p*-Value	OR (95% CI)	*p*-Value
Microinvasion (present *vs*. absent)	1.453 (0.308, 1.869)	0.637		
Grade of DCIS (high *vs*. intermediate *vs*. low)	0.965 (0.376, 1.481)	0.942		
ER (positive *vs*. negative)	0.291 (0.087, 1.013)	0.053	0.789 (0.171, 3.637)	0.762
PR (positive *vs*. negative)	0.380 (0.117, 1.234)	0.107		
HER2 (3+ *vs*. 2+ *vs*. 1+ *vs*. 0)	0.912 (0.496, 1.677)	0.767		
Ki-67 (>30% *vs*. ≤30%)	1.570 (0.327, 7.549)	0.573		
Abnormal lymph node on US (present *vs*. absent)	11.143 (3.367, 36.878)	<0.001	17.05 (3.089, 94.104)	0.001
PDW/PCT (>52.85 *vs*. ≤52.85)	6.120 (1.570, 23.859)	0.009	6.910 (1.389, 34.385)	0.018

DCIS, ductal carcinoma in situ; ER, estrogen receptor; PR, progesterone receptor; PDW, platelet distribution width; PCT, platelet crit.

## Discussion

The major concern over preoperatively diagnosed DCIS *via* US-CNB is the risk of missing concomitant invasive carcinoma (21). In our study, we noted that 75 out of 260 patients (29.3%) preoperatively diagnosed with DCIS were confirmed with concomitant invasive carcinoma by postoperative pathology. This result was higher than the proportion reported by a meta-analysis (25.9%), which included 7,350 patients from 52 studies (5). The main reason for this may be that our study was focused on US-CNB, which was conducted on masses. However, according to previous studies, the mass itself implied an increased risk of infiltration (22).

DCIS most often presents as segmental or linearly distributed calcifications on mammography. It may be due to deposits of calcium caused by irregular necrosis in the center of lesions. In the study by Roger Jackman et al. on DCIS, pathological underestimation occurred in 35 out of 144 patients who presented with only masses, with an underestimation rate of 24.3%, while the same phenomenon occurred in 148 out of 1,182 patients who presented with only calcifications, with an underestimation rate of 12.5%. The former was 1.9 times higher than the latter (23). A similar finding was confirmed in other two studies by King et al. and Kondo et al. (24, 25). It appeared that underestimation of invasive carcinoma occurred more in masses than in calcifications. Coincidently, there were other studies revealing that invasive carcinomas usually manifested as masses without calcification; in other words, most carcinomas that presented only as a mass were infiltrative, and this percentage could be as high as 84% (26, 27). Our study revealed that among patients diagnosed with DCIS preoperatively, those with linear or segmental distributed calcification on mammography were less likely to be diagnosed with invasive carcinoma as compared with those without linear or segmental distributed calcification on mammography. This result was consistent with previous studies.

Microinvasion is considered to be a transitional stage in the development progress from DCIS to invasive ductal carcinoma (IDC) (15). Several studies have revealed its invasive and metastatic potential as well as its importance as a distinct entity from pure DCIS deserving attention (28). The relationship between microinvasion and pathological underestimation has also attracted the attention of investigators. In the study by Park et al., presence of microinvasion in core needle biopsy was an important risk predictor for occurrence of pathological underestimation (29). Doria et al. reached the same conclusion in their study. They went on to include microinvasion in their prediction model and achieved good performance (30). Van la Parra et al. found that patients diagnosed with concomitant microinvasion in DCIS preoperatively not only were more likely to be diagnosed with invasive carcinoma postoperatively but also had a significantly increased risk of ALN metastasis (31). Similarly, in our study, presence of microinvasion was an important predictor for concomitant invasive carcinoma, but it was not associated with ALN metastasis.

There were studies reporting that sensitivity of preoperative axillary ultrasonography for lymph node metastasis ranges from 35% to 95% (32, 33). The main reason for this discrepancy was the subtle difference in diagnostic criteria of abnormal lymph node among different centers (34). In our study, lymph nodes would be considered abnormal if they met one or more of the following criteria: irregular shape, thickness ≥3 mm, absent or small core, and irregular blood flow from outside the core. Our findings suggested that patients with preoperative abnormal lymph node on ultrasound had a higher risk of being diagnosed with invasive carcinoma and developing lymph node metastasis postoperatively. Likewise, in the study by Chang et al., presence of abnormal lymph node on ultrasound implied a higher risk of pathological underestimation (18). In their study, abnormal lymph nodes referred to those which were round in shape and/or had hypoechoic cortical thickening (wall thickness >2 mm).

In contrast to IDC, DCIS cells are restricted to the ductal system of the breast and theoretically are incapable of metastasizing to ALNs. However, in our study, five patients who were diagnosed with pure DCIS postoperatively presented with lymph node metastasis (metastasis area >2 mm), accounting for 2.7% of all patients with pure DCIS. The same phenomenon was also reported in previous studies with chance of occurrence from 0.98% to 13% (35, 36). Investigators attributed it to microinvasion in the excision specimen that could not be detected, which, in other words, was another kind of pathological underestimation, i.e., a gap between the limit of modern medical testing and the actual biological behaviors (37). We have identified abnormal lymph node on ultrasound as an important predictor not only for concomitant invasive carcinoma but also for ALN metastasis. In addition, we noted another hematological index PDW/PCT, which was also related to metastasis of ALNs.

PDW was an index indicating variation in platelet volume, and PCT was another index indicating the percentage of platelets in total blood. In contrast, PDW/PCT provided a better response to changes in platelet count and activity (38, 39).

The interaction between platelets and tumor cells was non-ignorable in occurrence and metastasis of tumor cells. Chemokines and pro-inflammatory factors released by growing tumor cells could promote production, aggregation, and activation of platelets (40). In return, activated platelets had an effect on tumor cells by boosting their abilities of infiltration and migration, as well as enhancing their survival ability in the circulatory system (41).

In addition, a large number of factors secreted by activated platelets, such as vascular endothelial growth factor (VEGF) and platelet-derived growth factor (PDGF), were able to promote both angiogenesis and lymph angiogenesis. It has been reported that VEGF-A and VEGF-C were able to promote tumor metastasis by promoting lymph angiogenesis, while PDGF could promote the release of VEGF from fibroblasts in the microenvironment (42–44). Meanwhile, VEGF-C could act on megakaryocytes and lead to a change in platelet count (45). This complex interplay between tumor cells, platelets, and microenvironment ultimately resulted in an enhanced ability of tumor cells to metastasize through lymphatic system. And this series of changes could be well reflected by the index PDW/PCT.

PDW/PCT has been shown to correlate with prognosis of breast cancer patients. In the study by Takeuchi et al., higher PDW/PCT (>59.0) was related to shorter disease-free survival (DFS) (39). And in our study, patients with high PDW/PCT (>52.85) had an increased risk of ALN metastasis.

To our surprise, some risk predictors that have been identified in other studies, such as size and palpability of the lesion, pain, and number of puncture strips, did not show positive results in our study (46, 47). The reason may be that some of these predictors have been reported in previous articles and effective preventive measures have been taken by clinicians, while others may be caused by Chinese low breast cancer screening rate. As for pathological indicators, although a few studies on HER2 and ER have yielded positive results, their value needs to be demonstrated by more large-scale clinical trials (10, 18).

Due to immutable objective conditions, there are some limitations in this study. First, it has been reported that the type of core needles was also an important predictor, while in the present study, we could not analyze its predictive value because all patients underwent biopsies with a 14-gauge semi-automated core needle. Second, we were unable to evaluate impact of ALN metastasis on prognosis of patients with preoperatively diagnosed DCIS due to the lack of follow-up data. Third, our study was based on a single center with a limited sample size. Thus, our findings need to be validated by more in-depth and multicenter clinical trials.

## Conclusion

For patients with a preoperative diagnosis of DCIS *via* US-CNB, presence of microinvasion, presence of abnormal lymph node on ultrasound, and absence of linear or segmental distributed calcification on mammography imply an increased risk of concomitant invasive carcinoma, while presence of abnormal lymph node on ultrasound and increased PDW/PCT (>52.85) imply a higher risk of ALN metastasis. For such patients, appropriate ALN management is necessary.

## Data Availability Statement

The raw data supporting the conclusions of this article will be made available by the authors, without undue reservation.

## Ethics Statement

The studies involving human participants were reviewed and approved by Ethics Committee of China Medical University (Approval number: AF-SOP-07-1.1-01). The patients/participants provided their written informed consent to participate in this study. Written informed consent was obtained from the individual(s) for the publication of any potentially identifiable images or data included in this article.

## Author Contributions

FJ was the director for the fund and conceived this study. YL and XW collected medical records and drafted manuscript. AZ, XY, and ZJ assisted in revising the manuscript. All authors contributed to the article and approved the submitted version.

## Funding

This study was supported by the National Natural Science Foundation of China (No. 82073282).

## Conflict of Interest

The authors declare that the research was conducted in the absence of any commercial or financial relationships that could be construed as a potential conflict of interest.

## Publisher’s Note

All claims expressed in this article are solely those of the authors and do not necessarily represent those of their affiliated organizations, or those of the publisher, the editors and the reviewers. Any product that may be evaluated in this article, or claim that may be made by its manufacturer, is not guaranteed or endorsed by the publisher.

## Abbreviations

DCIS, ductal carcinoma *in situ*; ALND, axillary lymph node dissection; IDC, invasive ductal carcinoma; PR, progesterone receptor; SLNB, sentinel lymph node biopsy; BCS, breast-conserving surgery; US-CNB, ultrasound-guided core needle biopsy; HER2, human epidermal receptor 2; PLR, platelet–lymphocyte ratio; SII, systemic immune infiltration index; PDW, platelet distribution width; ER, estrogen receptor; NLR, neutrophil–lymphocyte ratio.
